# Supplementary material for: Critical Illness in Migrant Workers in the Windsor-Essex Region: A Descriptive Analysis
Source: Int J Environ Res Public Health. 2023 Aug 16;20(16):6587. doi: 10.3390/ijerph20166587 (PMC10454922; doi:10.3390/ijerph20166587)
Supplement: Supplementary file 1 [file ijerph-20-06587-s001.zip › ijerph-2521640-supplementary.pdf]

Supplementary Materials

INTERVIEW QUESTIONS FOR MIGRANT WORKERS WITH CRITICAL ILLNESS

1. How long have your worked on your current farm?
2. What other farms have you worked on in Canada?
    1. When did you work on these farms?
3. Which farm were you working on when you became ill?
4. Do you want to share your experience of your employer when you became ill?
    1. Were they helpful?
    2. How did they help you?
    3. Can you think of a way that they could have been more helpful?
5. Were you worried you would lose your job when you became ill?
6. Were you worried you would be deported if you were found to be very ill?
7. Did you lose your job when you became ill?
8. Did you know that your health care was free when you became ill?
9. Did you know where to go for help when you became ill?
10. What insurance did you have?
11. In your own words, can you tell us what happened?
    1. When did you first think you were ill?
    2. Who brought you to the doctor?
    3. who supported you emotionally ?
    4. Who supported you financially?
    5. what role did your employer have in your experience?
    6. what role did the embassy have in your experience?
    7. How did the social worker help, or not help you?
    8. How did the doctor help, or not help you?
    9. Were there other health care workers that helped you?
    10. Were there other health care workers who you felt did not help you?
12. Did you seek legal support?
    1. Did you know where to go for this?
13. Did you have financial challenges ?
    1. Can you describe them?
14. What challenges did you have in healthcare coverage?
15. Did you feel alone in this battle?
    1. How did you reach out to people for support? (eg phone, Whats App)
16. What other formal and informal supports (i.e. family, friends, social worker, case worker, anything from back home?) they had access to and how often they contacted their supports.
17. In the future, what would they like to see changed, for people in their position (as critically ill migrant workers).
